# Effectiveness of 12-month GnRH antagonist treatment for endometriosis-associated pain: driven by reduction in uterine bleeding?

**DOI:** 10.1093/hropen/hoag031

**Published:** 2026-04-08

**Authors:** Thomas M D’Hooghe

**Affiliations:** Research Group Reproductive Medicine, Department of Development and Regeneration, Organ Systems, Group Biomedical Sciences, KU Leuven (University of Leuven), Leuven, Belgium; Department of Obstetrics, Gynecology and Reproductive Sciences, Yale School of Medicine, New Haven, CT, USA; Global Medical Unit Fertility, Research and Development, Merck Healthcare KGaA, Darmstadt, Germany

## Introduction

For the medical treatment of endometriosis-associated pain, the Guideline Development Group (GDG) in the most recent ESHRE endometriosis guidelines recommends (as a good practice point (GPP)) that clinicians take a shared decision-making approach and take individual preferences, side effects, individual efficacy, costs, and availability into consideration when choosing hormone treatments for endometriosis-associated pain (Becker *et al*., 2022). A strong recommendation is made to offer women one of the following hormonal treatment options: combined hormonal contraceptives (oral, vaginal ring, or transdermal), progestogens (systemic, a levonorgestrel-releasing intrauterine (LNG-IUS) system or an etonogestrel-releasing subdermal implant), GnRH agonists, or GnRH antagonists (Becker *et al*., 2022). The ESHRE GDG also recommends (as a GPP) that both GnRH agonists and antagonists are prescribed as second-line options (e.g. if hormonal contraceptives or progestogens have been ineffective) due to their side-effect profiles. While it is strongly recommended that clinicians should consider prescribing combined hormonal add-back therapy alongside GnRH agonist therapy to prevent bone loss and hypo-estrogenic symptoms, it is also emphasized that evidence is limited regarding dosage or duration of treatment with GnRH agonists or antagonists. Therefore, well-designed studies are needed to evaluate the effectiveness and safety of both GnRH agonists and antagonists in various doses, and after long-term treatment (beyond 6 months).

## Effectiveness and safety of treatment of moderate–severe endometriosis-associated pain with linzagolix for 12 months

In this issue of *Human Reproduction Open*, Donnez *et al*. (2026) provide an important and clinically relevant contribution to address this gap, evaluating if the efficacy of the GnRH antagonist linzagolix to reduce endometriosis-associated pain, observed after 6 months of treatment in the placebo-controlled Edelweiss phase III trial (Donnez *et al*., 2024), can be maintained after an additional 6 months (Months 6–12) of treatment. The co-primary endpoints were a reduction in dysmenorrhea (DYS) and non-menstrual pelvic pain (NMPP) at Month 12 compared to baseline (before initiation of linzagolix treatment in the Phase III trial). All subjects (n = 353) received either 75 mg linzagolix (LGX) alone or 200 mg combined with hormonal add-back therapy (ABT) once daily for the additional 6 months. Outcomes were assessed in all patients but are clinically relevant in the 239 patients who had received active treatment during the 6 months of the Phase III trial and then continued with the same treatment between Months 6 and 12. Pain was measured daily on a verbal rating scale and recorded in an electronic diary (e-diary).

By Month 12, the proportion of subjects with a significant reduction in DYS and stable or decreased use of analgesics was 55.9% in the 75 mg LGX group and 91.0% in the 200 mg+ABT LGX group. The proportion of subjects with a significant reduction in NMPP and stable or decreased use of analgesics was 59.5% in the 75 mg LGX group and 67.6% in the 200 mg+ABT LGX group (Donnez *et al*., 2026).

A steady reduction in mean daily dyschezia, dyspareunia, and overall pelvic pain scores was observed in both linzagolix groups from baseline to Month 12, with a more marked reduction in the 200 mg+ABT LGX group. The mean change from baseline for overall pelvic pain scores (NRS) at Month 12 was −3.46 (vs −2.92 at Month 6) in the 75 mg LGX group and −4.35 (vs −3.76 at Month 6) in the 200 mg+ABT LGX group. Quality of life, assessed using the EHP-30 questionnaire, showed clear improvements at Month 12 (total score reductions) in both LGX groups, especially in the 200 mg+ABT LGX group. Changes in BMI were minimal, while adverse events like hot flushes were encountered in fewer than 1% of subjects (Donnez *et al*., 2026).

Overall, this study (Donnez *et al*., 2026) is clinically relevant, as it provides new information on effectiveness and safety related to both the duration and dose of GnRH antagonist treatment of endometriosis-associated pain, identified as a gap in the latest endometriosis ESHRE guidelines (Becker *et al*., 2022). Indeed, treatment of women with moderate–severe endometriosis-associated pain with LGX for 12 months was shown to be effective and safe, particularly in the 200 mg+ABT LGX group. The results with 75 mg LGX alone, presumably selected based on a previous Phase 2A study (Donnez *et al*., 2020), were comparatively disappointing, but became significant at 6 and 12 months compared to placebo. While the 75 mg LGX dose could represent an option for patients reluctant to undergo ABT or in whom ABT is contraindicated, it would be interesting to explore the effectiveness of alternative doses (e.g. 100 mg) in future studies.

## Is pain relief under GnRH antagonist treatment driven by a reduction in uterine bleeding?

A number of considerations can be made to position the Donnez *et al*. (2026) study in a broader context.

First, out of 486 patients who completed the 6-month initial phase III study (Donnez *et al*., 2024), 353 (73%) participated in the 6-month extension study, and it is not clear why the other 27% declined participation for reasons not disclosed in the extension study (Donnez *et al*., 2026).

Second, the association between pain reduction and reduction in uterine bleeding under GnRH antagonist treatment requires further discussion. While the mean baseline numbers of days per month with uterine bleeding (including spotting) were around 7 days (75 mg LGX: 6.88 days; 200 mg+ABT LGX: 6.76 days), these durations steadily declined during treatment and at Month 12 were reduced to −2.62 days (vs −2.29 at month 6) and to −5.26 days (vs −5.03 at Month 6) for the 75 mg LGX and 200 mg+ABT LGX groups, respectively, implying that indeed at 12 months, the 75 mg LGX and 200 mg+ABT LGX groups still had 4 days and 1.5 days of uterine bleeding, respectively (compared to 4.5 days and 2.3 days at 6 months (Donnez *et al*., 2024)). Similarly, other oral GnRH antagonist therapies for endometriosis have been reported to decrease the number of bleeding days, i.e. increase the proportion of women with amenorrhea in a dose-dependent way to 28% (elagolix 150 mg once daily), 65% (elagolix 200 mg twice daily) after 6 months, and to 80% and 77% (extension study with relugolix CT (40 mg relugolix, 1 mg estradiol, and 0.5 mg norethisterone acetate) after 12 and 24 months, respectively) (Taylor *et al*., 2017; Becker *et al*., 2024).

I hypothesize that the higher effectiveness of higher versus lower doses of elagolix and linzagolix observed in placebo-controlled trials (Taylor *et al*., 2017; Donnez *et al*., 2024), confirmed in the current linzagolix extension study (Donnez *et al*., 2026), can be at least partially explained by the lower number of bleeding days/higher proportion of women with amenorrhea in the higher dose group. This hypothesis is indirectly supported by a meta-analysis (Muzii *et al.*, 2016) showing continuous oral contraceptive pill (OCP) use may be superior to cyclic OCP use for dysmenorrhea recurrence (Muzii *et al.*, 2016), without a difference in safety profile, which explains why the ESHRE endometriosis guidelines state that women suffering from endometriosis-associated dysmenorrhea can be offered the continuous use of a combined hormonal contraceptive pill (weak recommendation) (Becker *et al*., 2022).

## Can dysmenorrhea be assessed in the absence of menstruation?

Medical treatment for endometriosis-associated pain is based on hormonal suppression that reduces the number of uterine bleeding days, with variations according to the type and dose of medical treatment. It would be of clinical interest to address whether pain relief is more related to the reduction in the number of bleeding days and/or to a reduction in serum estrogen levels that suppresses the activity of endometriosis lesions. Unfortunately, this was not possible in the registration or extension studies for oral GnRH antagonist treatment for endometriosis-associated pain (Taylor *et al*., 2017; Giudice *et al*., 2022; Becker *et al*., 2024; Donnez *et al*., 2024; Donnez *et al*., 2026). This is because the co-primary endpoints of these studies, DYS and NMPP, were reported in a way that does not allow this assessment. DYS and NMPP scores were calculated by averaging daily e-diary answers to an endometriosis-related pelvic pain questionnaire over the corresponding 28 calendar days prior to the assessment point. Responses of ‘No pain’, ‘Mild pain’, ‘Moderate pain’, and ‘Severe pain’ were assigned a score of 0, 1, 2, and 3, respectively. DYS counted the number of days with uterine bleeding, defined as those on which the subject records any uterine bleeding or spotting in her e-diary; while NMPP counted the days with no uterine bleeding. If a subject’s mean score for DYS was undefined numerically because her daily e-diary indicated that she did not experience any uterine bleeding at all during the 28-day calendar period, her mean score for DYS was set equal to zero (reflecting the absence of any DYS during that time period). Similarly, if a subject’s diary reports showed that she only experienced days with uterine bleeding during the period, then the mean score for NMPP was set equal to zero. However, if there are no days of uterine bleeding in a given month, then there is no menstruation, and therefore it is simply not possible to assess the score for dysmenorrhea, as dysmenorrhea is defined as pelvic pain during menstruation, and hence a mean score of zero for DYS is not appropriate. Similarly, if a given month includes only days of uterine bleeding, then it is simply not possible to assess the score for NMPP, as there was only uterine bleeding, no days without uterine bleeding, and hence a mean score of zero for NMPP is not appropriate. While fully recognizing that the definition and calculation of these endpoints was provided by regulatory authorities insisting on a reduction in pain for the two co-primary endpoints of DYS and NMPP individually, it is clinically more relevant to assess the overall reduction in overall pelvic pain on a daily basis, and to evaluate the association between daily pain scores and the presence/degree or absence of uterine bleeding. Current e-diaries, digital technology, and artificial intelligence (AI) approaches can be used to enable the analysis of such patient-reported assessments. These would allow testing of the hypothesis that days of uterine bleeding are more associated with pelvic pain than days without uterine bleeding, and to consider other relevant variables that may co-determine pain perception (amount of uterine bleeding, serum estrogen levels, medication dose, etc.). This is important to better understand the relationship between endometriosis-associated pain and uterine bleeding overall and at an individual patient level. In addition, it would help us better understand how hormonal medical treatment for endometriosis-associate pain is effective: either only through reducing the number of uterine bleeding days and the amount of uterine bleeding (representing endometrial bleeding, which may be considered as a proxy for bleeding/metabolic activity in endometriosis lesions), and/or through other direct effects that suppress the activity of endometriosis lesions.

## Conclusion

The GnRH antagonist linzagolix can be used safely and effectively for 12 months for women with moderate to severe endometriosis-associated pain and represents a valid second-line treatment. However, the study design typically used for the evaluation of GnRH antagonists in registration trials is based on monthly averages of daily pain scores for DYS or NMPP and does not allow the determination of the extent to which their effectiveness can be explained by a reduction in the number/intensity of uterine bleeding. Daily rating of both pelvic pain scores and absence/presence/degree of uterine bleeding, using digital technology and advanced (potentially AI driven) analytics is recommended.

## Author’s roles

T.M.D.’H., as the sole author, is fully responsible for the ideas, concepts, hypothesis, and solutions for a more refined assessment of both pain and uterine bleeding as relevant outcomes of medical hormonal treatment of endometriosis-associated pain.

## Funding

No funding was provided for the content presented in this commentary.

## Conflict of interest

T.M.D.’H. is a Guest Professor in Reproductive Medicine, Department of Development and Regeneration, University of Leuven (KU Leuven), Belgium, and Professor Adjunct, Department of Obstetrics Gynecology, Yale University, New Haven, USA. He also serves as Editor-in-Chief of Gynecologic and Obstetric Investigation. Since October 2015, he has served as Vice-President in the Medical Unit Fertility, Research and Development, Merck KGaA, Darmstadt, Germany (2025-1/2026: Head of Global Medical Affairs Fertility; since 2/2026: Senior Executive Medical-Scientific Director Fertility). His role in this publication is part of his academic work; he does not regard this as a conflict of interest as Merck KGaA is not involved.
